# Dietary Changes During COVID-19 Lockdown in Adults With Type 1 Diabetes on a Hybrid Artificial Pancreas

**DOI:** 10.3389/fpubh.2021.752161

**Published:** 2021-10-27

**Authors:** Claudia Vetrani, Ilaria Calabrese, Silvia Di Rienzo, Mariasofia Pagliuca, Annamaria Rivieccio, Raffaele De Angelis, Gabriele Riccardi, Angela Albarosa Rivellese, Giovanni Annuzzi, Lutgarda Bozzetto

**Affiliations:** Department of Clinical Medicine and Surgery, Federico II University, Naples, Italy

**Keywords:** type 1 diabetes, diet, eating habits, diet composition, glucose control, COVID-19, lockdown, hybrid artificial pancreas

## Abstract

In this retrospective analysis, we examine the impact of the lockdown of the coronavirus pandemic (COVID-19) on eating habits in individuals with type 1 diabetes (T1D) on a hybrid artificial pancreas (HAP). Dietary composition before and during lockdown was assessed by 7-day food records of 12 participants with T1D on HAP (three men and nine women, ages 38 ± 13 years, HbA1c 6.8 ± 0.3%, M ± SD). Continuous glucose monitoring (CGM) metrics and lifestyle changes (online questionnaire) were also assessed. Compared to prelockdown, reported body weight tended to increase during lockdown with no changes in total energy intake. Participants significantly decreased animal protein intake (−2.1 ± 3.7% of total energy intake, *p* = 0.048), but tended to increase carbohydrate intake (+17 ± 28 g/day, *p* = 0.052). These changes were induced by modifications of eating habits at breakfast and lunch during weekdays. Patients consumed more cereals (+21 ± 33 g/day, *p* = 0.038), whole grain (+22 ± 32 g/day, *p* = 0.044), and sweets (+13 ± 17 g/day, *p* = 0.021), and less animal protein sources (−42 ± 67 g/day, *p* = 0.054). Participants showed a more regular meal timing and decreased physical activity. Blood glucose control remained optimal (time-in-range 76 ± 8 vs. 75 ± 7% before lockdown), and daily total insulin infusion increased (42 ± 10 vs. 39 ± 12 I.U., *p* = 0.045). During the lockdown, patients with T1D on HAP modified dietary habits by decreasing animal protein and increasing carbohydrate intake. This increase, mainly concerning whole grain and low-glycemic-index products, did not influence blood glucose control.

## Introduction

The coronavirus pandemic (COVID-19) yielded a lockdown period in many countries to limit the spread of the virus. In Italy, it started on March 9, 2020, and lasted until May 3, 2020. Lockdown rules did not allow leaving home except for specific reasons (health, work, and shopping for basic needs) with a withdrawal of all non-essential services. Such measures translated into self-isolation and social distancing deeply affecting the lifestyle and behaviors of individuals.

These lifestyle changes, concerning physical activity, stress, and nutrition, and the lack of access to outpatient diabetes clinics, apart from interacting with their diabetes team by teleconsulting, were likely to adversely affect blood glucose control in patients with type 1 diabetes (T1D). However, in a large cohort of patients with T1D, an improvement in blood glucose control during lockdown was observed, which was related to lifestyle changes, including more regular eating habits, evaluated by a qualitative online questionnaire (1).

The possible role of dietary changes on this improvement is not known. Diet composition is a key determinant of blood glucose control in individuals with T1D (2, 3), over specific features of the patient (physical activity, insulin doses, illness, stress, pain, dehydration, and menstrual period) (4). Moreover, it has been shown that the time spent in the optimal range of blood glucose concentration after meals, especially lunch and dinner, strongly predicts daily blood glucose control (5).

Foods containing fiber and/or those with a low glycemic index induce a better metabolic profile (6, 7). In addition, others food components, such as fats and proteins, can affect blood glucose control (8, 9). Quantitative and qualitative changes in eating habits are linked to several factors that could have acted during the lockdown. First, food access and availability can drive the food choices of an individual (10), whereas emotional conditions, i.e., stress, sadness, fear, and anxiety are known to influence dietary patterns and quality of the diet (11).

Changes in eating behaviors during the pandemic have been reported in the general population (12–14) and also in patients with T1D (15). As expected, increased consumption of comfort foods, in particular sweets, was reported. To the best of our knowledge, no studies reporting nutritional composition are available so far.

Therefore, hypothesizing that changes in diet composition could also have contributed to the observed improvement in glucose control, we evaluated the effects of COVID-19-related confinement on dietary habits and nutritional composition in individuals with T1D treated with a hybrid artificial pancreas (HAP).

## Materials and Methods

This retrospective analysis was conducted in compliance with the Strengthening the Reporting of Observational Studies in Epidemiology (STROBE) statement checklist (16).

### Participants

Individuals with T1D with a HAP (MiniMed 670G®) attending the Diabetes Outpatient Clinic of Federico II University Hospital (Naples, Italy) and regularly filling in food records during their follow-up were screened for eligibility. All patients with a 7-day food record completed both before (January–February 2020) and during (March–April 2020) lockdown were included in this study. For the use of her/his data, each participant gave informed consent following the approval of the Ethical Committee of the Federico II University.

### Methods

Patients were provided with a 7-day food diary along with instructions including descriptive information for identifying foods eaten and guidelines for calculating portion size for various foods. They were asked to record all foods and drinks consumed, including dressing, reporting portions by household measures (cup, spoons, etc.) or weight, and providing as many details as possible (i.e., cooking methods and brands names). Food records were discussed with a skilled dietitian to check potential mistakes and missing information. Energy intake and dietary composition and food group consumption were calculated using the MetaDieta software (Meteda s.r.l., Ascoli-Piceno, Italy).

Qualitative lifestyle data were collected through a not-validated online questionnaire (1), evaluating changes between before and during the lockdown in physical activity (type and frequency), eating habits (food amount, meal timing, and the number of snacks), and body weight (Supplementary Table S1).

Blood glucose control was evaluated by the following metrics obtained through subcutaneous continuous glucose monitoring (CGM) (17): time-in-target range (TIR) (3.9–10.0 mmol/L), time-above-target range (TAR, >10.0 mmol/L and >13.9 mmol/L), time-below-target range (TBR, <3.9 mmol/L and <3.0 mmol/L) which were expressed as a percentage (%) of all CGM readings, mean glucose, and glycemic variability which was expressed by the coefficient of variation (CV%).

### Statistical Analysis

Data were expressed as mean ± SD unless otherwise stated. Differences between before and during lockdown were assessed by a paired sample *t*-test or Wilcoxon signed-rank test when parameters were not normally distributed. Normal distribution was checked using the Shapiro–Wilk test. A two-side *p* < 0.05 was considered significant. The statistical analysis was performed according to standard methods using the SPSS software version 25 (SPSS/PC; SPSS, Chicago, IL, USA).

## Results

All patients with T1D with a HAP were screened for eligibility (*n* = 22). Ten patients were excluded due to: no filling of 7-day food records before lockdown, incomplete dietary data registration, daily energy intake <800 kcal, or problems with the CGM sensor. Therefore, 12 participants (three men and nine women, aged 38 ± 13 years, BMI 25 ± 4 kg/m^2^, HbA1c 6.8 ± 0.3%, and diabetes duration 19 ± 9 years) met the inclusion criteria and were included in the analysis. On average, food records were completed 28 ± 9 days after the beginning of lockdown.

### Dietary Composition

Dietary energy intake did not differ significantly between before and during lockdown (Table 1). During the lockdown, participants decreased protein intake (17 ± 2% of daily total energy intake vs. 20 ± 3% before lockdown, *p* = 0.045), particularly animal protein (10 ± 3% of daily total energy intake vs. 13 ± 3% before lockdown, *p* = 0.048) (Table 1). In addition, a non-significant increase in carbohydrate amount was observed (177 ± 50 g/day vs. 159 ± 35 g/day before lockdown, *p* = 0.052) (Table 1). These changes were mainly triggered by modifications of dietary habits during weekdays, as shown in Table 2, with the reduction in total and animal protein intakes being significant on weekdays but not on weekends.

**Table 1:** Daily energy intake and dietary composition obtained through 7-day food records completed before and during the lockdown in the type 1 diabetes (T1D) study participants (*n* = 12).

	**Before lockdown**	**During lockdown**	***P***
Energy (kcal)	1,302 ± 317	1,347 ± 337	0.477
Protein (g)	61 ± 12	56 ± 12	0.229
(% TEI)	19 ± 3	17 ± 2	0.045
-Animal (g)	40 ± 9	34 ± 7	0.132
(% TEI)	13 ± 3	10 ± 3	0.048
-Plant (g)	20 ± 6	19 ± 6	0.689
(% TEI)	6.1 ± 1	6.3 ± 1	0.146
Carbohydrate (g)	159 ± 35	177 ± 50	0.052
(% TEI)	48 ± 5	51 ± 5	0.119
-Sugar (g)	44 ± 16	46 ± 16	0.627
(% TEI)	14 ± 4	13 ± 5	0.965
-Starch (g)	104 ± 26	115 ± 41	0.144
(% TEI)	33 ± 5	34 ± 6	0.303
Fat (g)	46 ± 10	48 ± 15	0.757
(% TEI)	33 ± 4	32 ± 5	0.724
-SFA (g)	13 ± 3	13 ± 5	0.658
-MUFA (g)	22 ± 5	20 ± 4	0.621
-PUFA (g)	5.8 ± 1.7	6.2 ± 2.0	0.667
Cholesterol (mg)	192 ± 51	155 ± 52	0.091
Fiber (g)	15 ± 6	15 ± 5	0.813
Alcohol (g)	0.5 ± 1.1	0.3 ± 0.9	0.679
Glycemic index (%)	54 ± 6	55 ± 7	0.475
Glycemic load	86 ± 24	97 ± 33	0.064

*M ± SD. MUFA, monounsaturated fatty acids; PUFA, polyunsaturated fatty acids; SFA, saturated fatty acids; TEI, total energy intake*.

**Table 2:** Daily energy intake and dietary composition on weekdays and weekends obtained through 7-day food records completed before and during the lockdown in the T1D study participants (*n* = 12).

	**Weekdays**			**Weekend**		
	**Before lockdown**	**During lockdown**	***P***	**Before lockdown**	**During lockdown**	***P***
Energy (kcal)	1,259 ± 204	1,299 ± 360	0.638	1,311 ± 369	1,339 ± 278	0.831
Protein (g)	64 ± 11	56 ± 13	0.077	58 ± 18	57 ± 11	0.850
(% TEI)	20 ± 3	17 ± 3	0.022	18 ± 5	17 ± 2	0.542
-Animal (g)	43 ± 10	33 ± 10	0.026	35 ± 14	38 ± 6	0.611
(% TEI)	14 ± 3	10 ± 3	0.010	9 ± 6	10 ± 5	0.532
-Plant (g)	20 ± 5	20 ± 7	0.721	21 ± 8	17 ± 7	0.105
(% TEI)	6 ±1	6 ± 2	0.866	5 ± 3	4 ± 3	0.062
Carbohydrate (g)	156 ± 34	175 ± 53	0.065	171 ± 55	179 ± 56	0.614
(% TEI)	47 ± 5	51 ± 7	0.106	49 ± 8	50 ± 7	0.741
-Sugar (g)	43 ± 16	46 ± 17	0.484	49 ± 20	47 ± 17	0.804
(% TEI)	13 ± 5	14 ± 6	0.784	14 ± 4	13 ± 4	0.554
-Starch (g)	102 ± 27	112 ± 44	0.193	112 ± 38	117 ± 43	0.650
(% TEI)	32 ± 6	34 ± 7	0.261	29 ± 14	28 ± 15	0.837
Fat (g)	46 ± 8	47 ± 17	0.920	48 ± 21	49 ± 12	0.925
(% TEI)	33 ± 4	31 ± 6	0.453	33 ± 8	33 ± 7	0.989
-SFA (g)	13 ± 3	13 ± 6	0.968	13 ± 3	15 ± 1	0.396
-MUFA (g)	22 ± 4	20 ± 5	0.340	22 ± 10	22 ± 6	0.998
-PUFA (g)	6 ± 2	6 ± 2	0.875	6 ± 2	6 ± 3	1.000
Cholesterol (mg)	201 ± 70	148 ± 61	0.070	179 ± 98	164 ± 61	0.693
Fiber (g)	16 ± 5	15 ± 6	0.918	16 ± 8	13 ± 6	0.309
Alcohol (g)	0.4 ± 1	0.3 ± 1	0.841	0.8 ± 2	0.1 ± 0.2	0.214
Glycemic index (%)	54 ± 6	54 ± 6	0.600	54 ± 5	56 ± 11	0.534
Glycemic load	84 ± 24	93 ± 35	0.117	93 ± 38	100 ± 38	0.496

*M ± SD. MUFA, monounsaturated fatty acids; PUFA, polyunsaturated fatty acids; SFA, saturated fatty acids; TEI, total energy intake*.

During the lockdown, dietary composition mainly changed at breakfast and lunch, whereas no significant changes were detected at dinner (Table 3). At breakfast, fat intake significantly increased during the lockdown, while animal protein and cholesterol decreased (Table 3). At lunch, patients consumed more carbohydrates and less protein, particularly animal proteins, while decreasing cholesterol intake and the glycemic index of the meal (Table 3).

**Table 3:** Energy intake and dietary composition of breakfast, lunch, and dinner obtained through 7-day food records completed before and during the lockdown in the T1D study participants (*n* = 12).

	**Breakfast**			**Lunch**			**Dinner**		
	**Before lockdown**	**During lockdown**	***P***	**Before lockdown**	**During lockdown**	***P***	**Before lockdown**	**During lockdown**	***P***
Energy (kcal)	172 ± 96	175 ± 130	0.771	556 ± 187	541 ± 196	0.569	534 ± 242	574 ± 261	0.256
Protein (g)	12 ± 8	16 ± 3	0.350	29 ± 13	24 ± 13	0.015	31 ± 15	27 ± 14	0.122
(% TEI)	20 ± 7	22 ± 4	0.803	18 ± 7	16 ± 6	0.039	22 ± 9	19 ± 9	0.088
-Animal (g)	7.9 ± 3.2	7.3 ± 4.4	0.200	14 ± 13	11 ± 12	0.046	21 ± 14	18 ± 12	0.138
(% TEI)	17 ± 9	11 ± 9	<0.001	10 ± 8	7 ± 7	0.019	15 ± 9	13 ± 10	0.293
-Plant (g)	1.4 ± 2.1	1.3 ± 2.3	0.841	12 ± 6	11 ± 6	0.210	7.7 ± 4.6	7.6 ± 4.8	0.833
(% TEI)	2.9 ± 4.3	3.6 ± 7.6	0.543	8.2 ± 3.3	8.2 ± 3.6	0.972	5.7 ± 3.3	5.7 ± 3.0	0.934
Carbohydrate (g)	31 ± 16	33 ± 19	0.092	71 ± 23	73 ± 26	0.544	64 ± 32	74 ± 43	0.077
(% TEI)	54 ± 10	55 ± 16	0.771	49 ± 13	53 ± 13	0.040	44 ± 17	48 ± 16	0.105
-Sugar (g)	14 ± 8	15 ± 10	0.091	16 ± 12	14 ± 12	0.120	16 ± 11	20 ± 13	0.060
(% TEI)	30 ± 9	31 ± 6	0.671	11 ± 12	11 ± 9	0.694	12 ± 14	11 ± 9	0.563
Fat (g)	9.1 ± 7.0	12 ± 8.6	0.032	23 ± 12	21 ± 12	0.181	21 ± 13	22 ± 16	0.918
(% TEI)	25 ± 7	29 ± 10	0.034	33 ± 10	31 ± 11	0.291	34 ± 14	32 ± 13	0.389
-SFA (g)	2.5 ± 2.0	2.8 ± 3.1	0.290	4.7 ± 3.7	4.3 ± 4.1	0.527	5.6 ± 4.9	6.3 ± 6.1	0.407
-MUFA (g)	2.1 ± 1.7	2.3 ± 1.7	0.425	11 ± 5	9 ± 5	0.063	10 ± 6	9 ± 6	0.203
-PUFA (g)	0.8 ± 0.7	1.0 ± 0.8	0.069	3.0 ± 2.0	2.5 ± 1.7	0.070	2.6 ± 1.8	2.8 ± 2.9	0.682
Cholesterol (mg)	24 ± 20	19 ± 18	0.092	69 ± 80	46 ± 61	0.041	110 ± 121	82 ± 96	0.151
Fiber (g)	1.7 ± 1.6	2.2 ± 2.1	0.126	7.8 ± 4.6	6.8 ± 4.0	0.124	6.7 ± 4.4	6.7 ± 3.9	0.950
Glycemic index (%)	48 ± 14	49 ± 14	0.692	51 ± 11	48 ± 10	0.023	58 ± 14	62 ± 14	0.340
Glycemic load	16 ± 9	29 ± 6	0.115	36 ± 13	35 ± 16	0.827	38 ± 23	45 ± 32	0.077

*M ± SD. MUFA, monounsaturated fatty acids; PUFA, polyunsaturated fatty acids; SFA, saturated fatty acids; TEI, total energy intake*.

**Figure 1:**
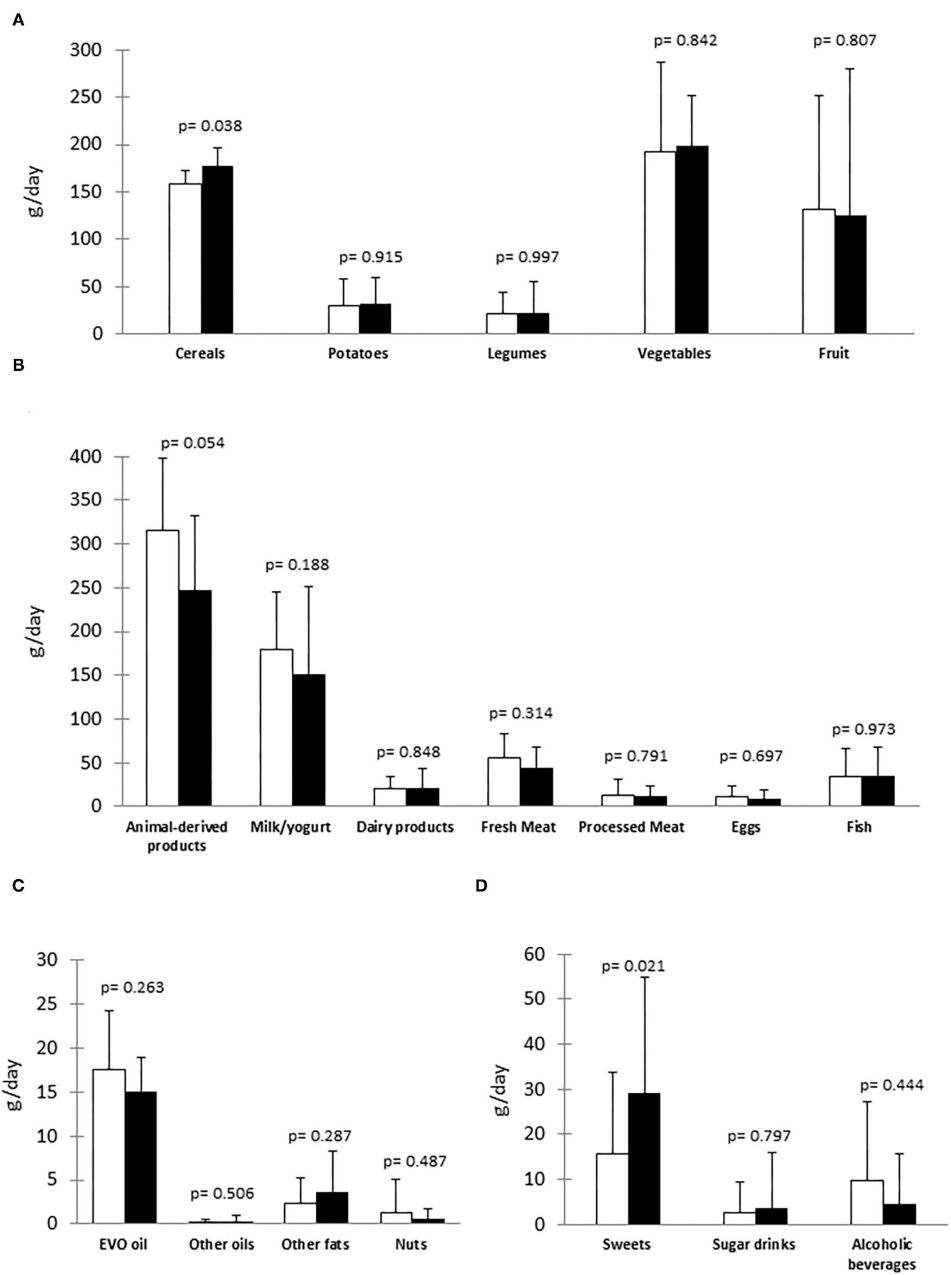
Food groups consumption before lockdown (White column) and during lockdown (Black column) in the type 1 diabetes (T1D) study participants (*n* = 12). **(A)** Main sources of carbohydrate/fiber; **(B)** Main sources of protein; **(C)** Main sources of fat; **(D)** Other food and beverages. EVO oil, extra-virgin olive oil.

Consumption of food groups is shown in Figure 1. During the lockdown, the patients significantly increased the consumption of cereals (177 ± 64 vs. 156±48 g/day before lockdown, *p* = 0.038, Figure 1A). This corresponded to an increase in whole grain products (157 ± 61 vs. 135±45 g/day, *p* = 0.044) with no changes in refined cereals (20 ± 23 vs. 22 ± 22 g/day, *p* = 0.728). The intake of pasta and rice increased during lockdown (404 ± 158 vs. 321 ± 165 g/week before lockdown, *p* = 0.038), whereas bread consumption did not change (463 ± 370 vs. 465 ± 281 g/week before lockdown, *p* = 0.970). The overall consumption of animal-derived products tended to decrease (247 ± 86 vs. 315 ± 83 g/day before lockdown, *p* = 0.054) with no major changes in the individual main sources of protein (Figure 1B). During the lockdown, the intake of sweets significantly increased (29 ± 26 vs. 16 ± 18 g/day before lockdown, *p* = 0.021, Figure 1D), and it mainly included chocolate and pastries (84 ± 7 1 vs. 24 ± 53 g/week before lockdown, *p* = 0.079). No changes were observed for other main food groups (Figures 1C,D).

### Blood Glucose Control

No significant differences were observed in the control of blood glucose between before and during the lockdown in TIR (75 ± 7 and 76 ± 8%, *p* = 0.652), TAR (26 ± 7 and 28 ± 9%, *p* = 0.608), TBR (3.8 ± 3 and 4.2 ± 3%, *p* = 0.746), and mean glucose (8.70 ± 1.0 and 8.36 ± 0.5 mmol/L, *p* = 0.420), respectively, with a tendency to reduced glycemic variability (CV%) during lockdown (30 ± 5 vs. 33 ± 4 before lockdown, *p* = 0.145).

Daily total insulin doses increased significantly during lockdown (42 ± 10 vs. 39 ± 12 I.U. before lockdown, *p* = 0.045), mainly due to an increase in basal infusion (23 ± 8 vs. 21 ± 7 I.U., *p* = 0.120).

### Lifestyle Changes

Data on lifestyle changes are reported in Supplementary Table S2. During the lockdown, seven participants reported a slight increase in body weight (+2 kg), three reported weight loss (−3 kg), and two reported no change at all. Ten participants reported a reduction in total physical activity. Eating habits were characterized by a more regular meal pattern in seven patients and no increase in snacking (no changes in 10 participants and a decrease in two participants).

## Discussion

We describe the impact of total lockdown on dietary habits in patients with T1D on a hybrid artificial pancreas. Our results indicate that lockdown for COVID-19 induced small but relevant modifications in dietary habits. In brief, a reduction of protein, particularly animal protein, and an increase in carbohydrate intake were detected. As confirmed by the evaluation of food groups consumption, these changes were mainly due to a reduction in the intake of overall animal sources of protein and an increase in the intake of whole grain cereals. They are partially in line with the current nutritional recommendations for a healthier dietary pattern in patients with T1D to achieve good blood glucose control (2, 3). On the other hand, during the lockdown, patients increased the consumption of sweets (mainly chocolate and pastries) around dinner time by 13 g. It is of note that these changes were in line with those observed in the general Italian population (14) and in patients with diabetes (15).

During the lockdown, eating habits were characterized by a more regular meal timing and snacking pattern that are considered as key features of a healthier dietary pattern, especially in individuals with T1D (18). We hypothesize that these changes in dietary habits were mainly related to the increased time spent at home which induced patients to consume cooked meals (i.e., meals including pasta and rice) rather than sandwiches or toasts, which would have resulted in higher consumption of foods containing animal protein. This is in line with the higher amount of carbohydrates consumed at lunch from foods with a lower glycemic index.

During confinement, participants slightly increased their body weight, likely due to the reduced physical activity, while the changes in daily energy intake were not statistically significant. In this cohort of patients with T1D on a HAP, blood glucose control did not change significantly during the lockdown, as it was expected considering the high performances of this insulin infusion system in keeping blood glucose in the optimal range. Furthermore, a trend to reduced glucose variability was observed. It is important to underline that blood glucose control was maintained through an increased basal insulin infusion, which is in line with impairment of insulin sensitivity due to decreased physical activity.

Our study has some strengths, particularly in relation to the use of the weighted 7-day food records that represent the gold standard for evaluating eating habits at an individual level (19). Additionally, lockdown for COVID-19 provided a unique opportunity to evaluate the effects of home confinement in a free-living T1D population on HAP.

A limitation of this study is the small sample size, which may have reduced its statistical power, impeding to detection of possible changes. Another limitation is the possible underreporting that is common to all types of food recordings. However, we compared intraindividual changes and, therefore, the same potential mistakes could be expected on both occasions. In addition, food records were discussed with a skilled dietitian to check for potential errors. A further limitation includes lifestyle changes that were investigated by a simple non-validated questionnaire. This questionnaire had been specifically structured to retrieve information related to the unique context of the lockdown (1). As for generalizing the results of this study to a wider T1D population, it must be considered that they refer to a cohort living in Italy, characterized by specific nutritional habits and only concern patients on a hybrid artificial pancreas.

In conclusion, our results show that during lockdown for COVID-19, Italian patients with T1D on a HAP changed some of their eating habits with no major effects on blood glucose control. Overall, they experienced a more regular eating pattern that also included a potentially healthy reduction in the intake of animal protein sources and increased consumption of meals containing foods with a lower glycemic index. Therefore, increasing good quality carbohydrate sources may not lead to worsening glycemic control.

## Data Availability Statement

The raw data supporting the conclusions of this article will be made available by the authors, without undue reservation.

## Ethics Statement

The studies involving human participants were reviewed and approved by Ethics Committee Federico II. The patients/participants provided their written informed consent to participate in this study.

## Author Contributions

CV, IC, GA, and LB contributed to the design of the study and the analysis and interpretation of data. CV, IC, and LB wrote the first draft of the report. GR, AAR, and GA provided relevant intellectual contributions to the development of the report. SD, MP, AR, and RD collected and analyzed the data. GA is the guarantor of this study, had full access to all the data in the study, takes responsibility for the integrity of the data, and the accuracy of the data analysis. All authors provided substantial contributions to the acquisition of data, critically revised the report, and gave final approval of the version to be submitted for publication.

## Conflict of Interest

The authors declare that the research was conducted in the absence of any commercial or financial relationships that could be construed as a potential conflict of interest.

## Publisher's Note

All claims expressed in this article are solely those of the authors and do not necessarily represent those of their affiliated organizations, or those of the publisher, the editors and the reviewers. Any product that may be evaluated in this article, or claim that may be made by its manufacturer, is not guaranteed or endorsed by the publisher.
