# Supplementary material for: Dietary Changes During COVID-19 Lockdown in Adults With Type 1 Diabetes on a Hybrid Artificial Pancreas
Source: Front Public Health. 2021 Oct 27;9:752161. doi: 10.3389/fpubh.2021.752161 (PMC8578275; doi:10.3389/fpubh.2021.752161)
Supplement: Supplementary file 2 [file Data_Sheet_2.docx]

**Supplementary table S1.** On-line questionnaire for the collection of qualitative lifestyle changes during the lockdown compared to pre-lockdown**.**

| **Items** | **Questions** |
| --- | --- |
| Body weight | DURING THE RESTRICTIONS, body weight was **More/Less/Same?**  If changed, how many kg? **Kg** |
| Physical activity | BEFORE THE RESTRICTIONS, how did you reach the workplace/school? **Walk/Bike/Public transport/Car**  DURING THE RESTRICTIONS, how did you reach the workplace/school? **Walk/Bike/Public transport/Car**  BEFORE THE RESTRICTIONS, how many times a week or a day did you perform the following activities? **Run Outdoor/Tapis Roulant/Walk Outdoor/Byke Outdoor/ Cycle Indoor/Gym/Gym Indoor/Soccer/Walk the dog/Stairs/ Housekeeping/Any other** (If you have indicated more than one activity please specify the frequency for each one)  DURING THE RESTRICTIONS, how many times a week or a day did you perform the following activities? **Run Outdoor/Tapis Roulant/Walk Outdoor/Byke Outdoor/ Cycle Indoor/Gym/Gym Indoor/Soccer/Walk the dog/Stairs/Housekeeping/Any other** (If you have indicated more than one activity please specify the frequency for each one) |
| Food Intake | DURING THE RESTRICTIONS, did you change meal times? **Yes/no**  DURING THE RESTRICTIONS, meal time was **More regular/Less regular/Same**?  DURING THE RESTRICTIONS, did you eat **More/Less/Same**?  DURING THE RESTRICTIONS, did you consume **More snacks/Less snacks/Same**? |

**Supplementary table S2.** Questionnaire-derived lifestyle changes compared to pre-lockdown in the type 1 diabetes participants in the study (n=12).

| **Lifestyle items** | **Increase** | **Decrease** | **Same** |
| --- | --- | --- | --- |
| Body weight | 7 | 3 | 2 |
| Total physical activity | 0 | 10 | 2 |
| Food amount | 0 | 2 | 10 |
| -Regularity of mealtimes | 7 | 1 | 4 |
| -Number of snacks | 0 | 2 | 10 |

Data are expressed as number of participants
